# The Interplay Between Pediatric Atopic Dermatitis and Neurodevelopmental Disorders: Emerging Evidence, Mechanistic Insights, and Clinical Implications

**DOI:** 10.3390/ijms27209270

**Published:** 2026-10-19

**Authors:** Xiaoya Ma, Zhuo Chen, Alexander Anstey, Pengjie Wan, Dan Deng

**Affiliations:** 1 Department of Dermatology, Shanghai Children’s Medical Center, Shanghai Jiao Tong University School of Medicine, Shanghai 200127, China; maxiaoya20010814@163.com (X.M.); chenzhuo@scmc.com.cn (Z.C.); 2 Department of Dermatology, Musgrove Park Hospital, Somerset NHS Foundation Trust, Taunton TA1 5DA, UK; alex.anstey@doctors.org.uk; 3 Allergy and Immunology Diagnostic and Treatment Center, Shanghai Children’s Medical Center, Shanghai Jiao Tong University School of Medicine, Shanghai 200127, China

**Keywords:** atopic dermatitis, attention-deficit/hyperactivity disorder, autism spectrum disorder, neurodevelopmental disorders, neuroimmunomodulation

## Abstract

Atopic dermatitis (AD) is a common chronic inflammatory skin disease in children and is increasingly recognized as a systemic disorder with potential consequences beyond the skin. Growing evidence links pediatric AD to neurodevelopmental disorders, particularly attention-deficit/hyperactivity disorder (ADHD) and autism spectrum disorder (ASD), but the mechanisms and clinical implications remain incompletely understood. Systemic and neuroimmune inflammation, altered T-helper-cell and cytokine signaling, and genetic and epigenetic susceptibility may contribute to this association. Sleep disruption and psychosocial stress represent additional potential pathways linking skin inflammation with neurodevelopment. Stronger associations have been reported in children with severe or early-onset AD, multiple atopic comorbidities, and persistent sleep disturbance, highlighting the importance of disease heterogeneity. This narrative review integrates epidemiologic evidence and proposed mechanisms to examine the skin–brain axis in pediatric AD, with particular attention to high-risk phenotypes and implications for developmental surveillance and multidisciplinary management. Although current evidence supports an association, causality remains uncertain. Prospective studies integrating AD phenotypes, biomarkers, and neurodevelopmental assessments are needed to clarify these relationships and determine whether early effective AD treatment can modify long-term neurodevelopmental trajectories.

## 1. Introduction

Atopic dermatitis (AD) is the most common chronic inflammatory skin disease of childhood, with an estimated global prevalence of 15–25% [1,2]. Characterized by eczematous lesions, intense pruritus, and substantial psychosocial burden, AD is increasingly recognized as a systemic immune-mediated disorder [1,2]. Targeted treatments, including biologics that block interleukin (IL)-4 receptor α (IL-4Rα) and Janus kinase (JAK) inhibitors, have substantially improved disease control [1]. However, the comorbidity burden extends beyond the classical atopic march of asthma, allergic rhinitis, and food allergy to include non-atopic conditions [1].

Emerging epidemiologic and mechanistic evidence indicates that pediatric AD is linked to a higher likelihood of neurodevelopmental disorders (NDDs)—notably attention-deficit/hyperactivity disorder (ADHD) and autism spectrum disorder (ASD) [1,3,4,5]. A pooled analysis estimated the prevalence of ADHD among children with AD at 9%, compared with 4% in adults with AD [3], highlighting the relevance of this comorbidity in pediatric populations.

The proposed skin–brain connection links cutaneous inflammation with neurodevelopmental outcomes through immune dysregulation, potential genetic susceptibility, sleep disturbance, and psychosocial stress. This review integrates epidemiologic evidence, proposed mechanisms, and clinical implications of pediatric AD–NDD comorbidity, with an emphasis on developmental surveillance, multidisciplinary management, and research priorities. ADHD and ASD are the primary focus; related cognitive symptoms and psychiatric or behavioral outcomes are discussed as distinct aspects of the broader comorbidity burden.

## 2. Search Strategy and Selection Criteria

Literature relevant to this narrative review was identified through targeted searches of PubMed, with searches updated during manuscript revision, most recently on 13 October 2026. Search terms were selected to capture studies addressing both pediatric atopic dermatitis and neurodevelopmental outcomes, and included combinations of “atopic dermatitis”, “eczema”, “child”, “pediatric”, “infant”, “neurodevelopment”, “neurodevelopmental disorder”, “attention-deficit/hyperactivity disorder”, “ADHD”, and “autism spectrum disorder”.

The reference lists of key articles and reviews were also examined to identify studies not captured by the initial search, supplemented by literature known to the authors. Studies were considered eligible if they contributed epidemiologic evidence, mechanistic insight, or clinically relevant observations relating inflammatory skin disease in early life to neurodevelopmental outcomes. Pediatric studies were the main focus; adult studies and experimental studies were included where relevant to the proposed mechanisms or clinical context.

Only articles published in English were included. Final inclusion of references was guided by their scientific rigor, conceptual relevance, and contribution to understanding potential systemic and neuroimmune links between pediatric atopic dermatitis and neurodevelopmental disorders. The narrative selection process and restriction to PubMed and English-language publications may have introduced selection bias and limited coverage of the available evidence.

## 3. Epidemiology

**Figure 1:**
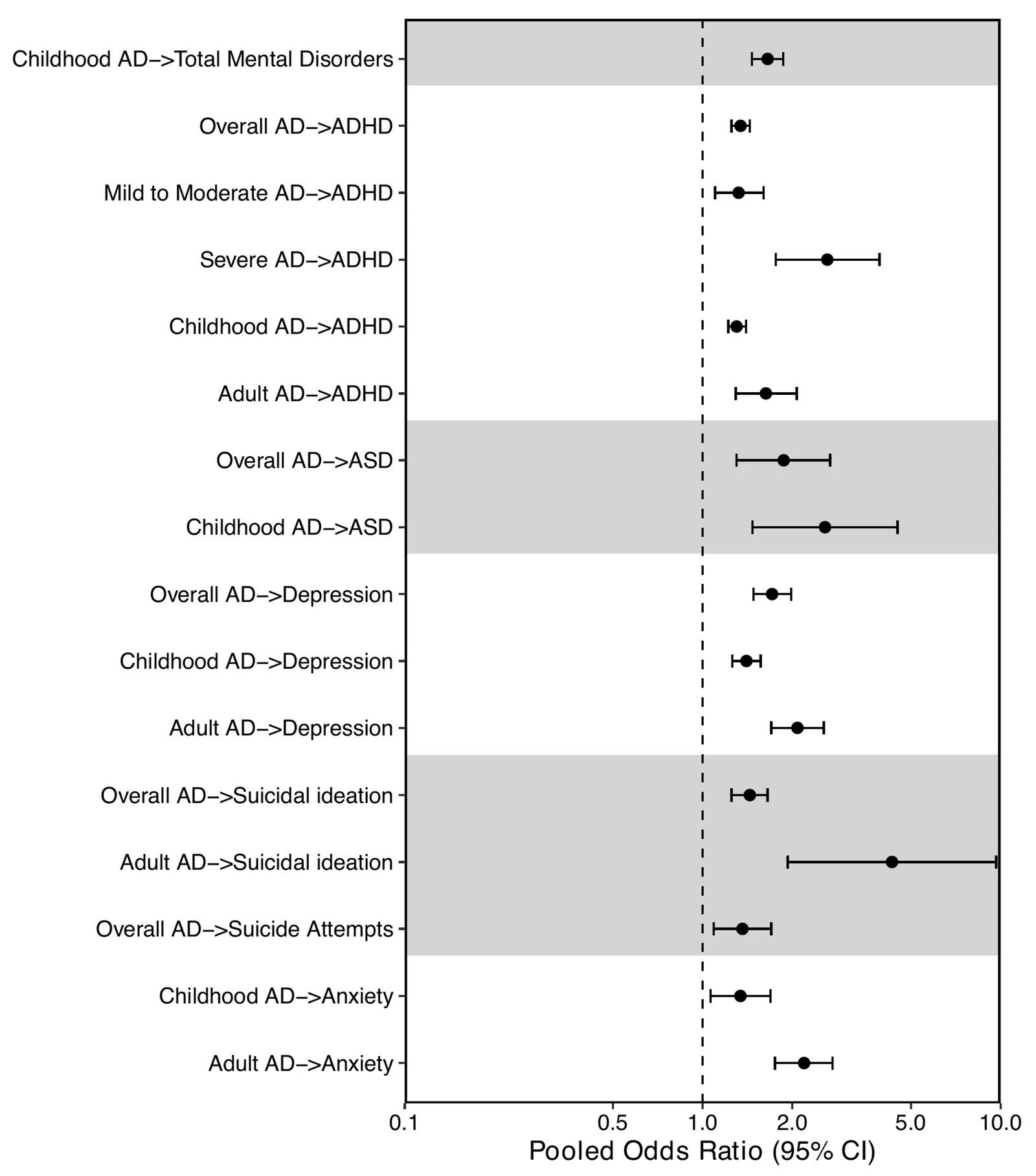
Risk of psychiatric and neurodevelopmental comorbidities in atopic dermatitis. Forest plot showing pooled odds ratios (ORs) with 95% confidence intervals (CIs) for psychiatric and neurodevelopmental comorbidities in individuals with atopic dermatitis (AD). Estimates are stratified by age group and disease severity and are derived from recent systematic reviews and meta-analyses. Alternating gray shading is used to visually distinguish different outcome categories. Outcomes include attention-deficit/hyperactivity disorder (ADHD), autism spectrum disorder (ASD), depression, anxiety, suicidal ideation, and suicide attempts. Data sources include childhood AD and total mental disorders [6]; ADHD, including severity- and age-stratified analyses [4]; overall ASD [7]; depression [8]; suicidality [9]; and adult depression, anxiety, and suicidal ideation [10]. Abbreviations: AD, atopic dermatitis; ADHD, attention-deficit/hyperactivity disorder; ASD, autism spectrum disorder; CI, confidence interval; OR, odds ratio.

Epidemiologic studies have associated pediatric AD with ADHD, ASD, and other neuropsychiatric outcomes. Age, disease severity, and atopic multimorbidity may influence the strength of these associations (Figure 1; Table 1).

### 3.1. Comorbidity Between AD and ADHD

An association between AD and ADHD has been reported in multiple large-scale epidemiologic studies. Systematic analyses indicate that the overall prevalence of ADHD among individuals with AD is approximately 7%, which is notably higher in pediatric populations (9%) than in adults (4%) [3].

Meta-analyses support an association between AD and ADHD [4,5]. Children with AD also exhibit more attention-deficit/hyperactivity symptoms in some studies [13], while children with ADHD have higher odds of AD [11]. These findings suggest a bidirectional association, with stronger estimates reported in European populations [4,14].

The strength of the AD–ADHD association may vary with disease severity. A meta-analysis reported particularly high odds of ADHD in severe AD (pooled OR 3.46; 95% CI, 2.25–5.30) relative to individuals without AD [7,15]. Stronger associations have also been reported when AD coexists with asthma or sleep disorders [4]. Early-onset AD has been associated with subsequent ADHD in some cohorts [16,17].

The burden of coexisting allergic disease may also influence the AD–ADHD association. The number of early-life atopic conditions has shown a dose-dependent relationship with ADHD risk [18,19]. Among U.S. children, food, respiratory, and skin allergies were associated with higher odds of ADHD after adjustment for multiple covariates [20]. Maternal AD has also been associated with a 15% higher risk of ADHD in offspring [21].

Findings are not uniform, however. A large UK population-based cohort reported no significant overall association between childhood AD and incident ADHD after adjustment for demographic characteristics, socioeconomic status, and other atopic conditions [22]. Differences in diagnostic ascertainment, including parental reports versus recorded clinical diagnoses, follow-up duration, severity definitions, and study populations may contribute to these discrepancies [4,16,22]. Healthcare utilization and shared familial factors may also influence observed associations. These differences limit generalization across all children with AD, while the observational designs preclude firm causal conclusions.

### 3.2. Comorbidity Between AD and ASD

Meta-analyses have reported an association between AD and ASD, particularly in pediatric populations [7,23]. AD has been associated with an approximately 1.6-fold higher risk of ASD in children compared with those without AD [6,24]. As with ADHD, the observed association may vary with allergic comorbidity, age at onset, and disease severity. Coexisting asthma or allergic rhinitis has been associated with higher ASD risk [19], while earlier onset and greater severity of infantile AD have been associated with subsequent ASD diagnosis [17]. In one cohort, infantile AD followed by later AD episodes and respiratory allergic disease was associated with an adjusted hazard ratio for ASD of up to 2.13 relative to the corresponding unexposed group [17].

Sex-specific findings have also been reported. A 2024 retrospective cohort study found a 9.8% higher risk of AD among males with ASD than in the general population, whereas no significant increase was observed among females with ASD [25]. These findings warrant further evaluation of sex-related differences in comorbidity patterns.

As in ADHD, a large UK population-based cohort found no significant overall association between childhood AD and incident ASD after adjustment for demographic characteristics, socioeconomic status, and other atopic conditions [22]. Substantial heterogeneity in meta-analyses [7,23] further suggests that AD phenotypes, study populations, diagnostic ascertainment, and follow-up influence observed estimates. The available evidence does not establish that AD-related immune activation causes ASD.

### 3.3. Other Neurodevelopmental, Cognitive, and Psychiatric Outcomes

Beyond ADHD and ASD, epidemiologic studies have reported associations of AD with additional neurodevelopmental and cognitive outcomes. A population-based case–control study in Taiwan found an association between AD and Tourette syndrome (TS), a neurodevelopmental tic disorder, and reported greater odds of TS with an increasing number of allergic comorbidities [26]. Pediatric AD has also been associated with caregiver-reported learning and memory difficulties [27]. In that study, the associations were primarily observed among children with coexisting neurodevelopmental conditions, particularly ADHD or learning disabilities. These findings highlight a potential additional cognitive burden in children with AD and neurodevelopmental comorbidities, although the measured symptoms do not establish diagnoses of intellectual disability or specific learning disorders.

The broader comorbidity burden of AD also includes emotional, behavioral, and psychiatric outcomes. Longitudinal pediatric evidence links AD with emotional and behavioral problems across childhood and adolescence [28], while systematic reviews have reported associations with depression, anxiety, and suicidality in pediatric, adult, or mixed-age populations [3,8,9,10]. Although distinct from NDDs, these outcomes provide important context for the broader mental health burden associated with AD, while findings from adult and mixed-age populations should be viewed as complementary rather than pediatric-specific evidence.

## 4. Comorbidity Mechanisms

### 4.1. Immunologic Mechanisms

**Figure 2:**
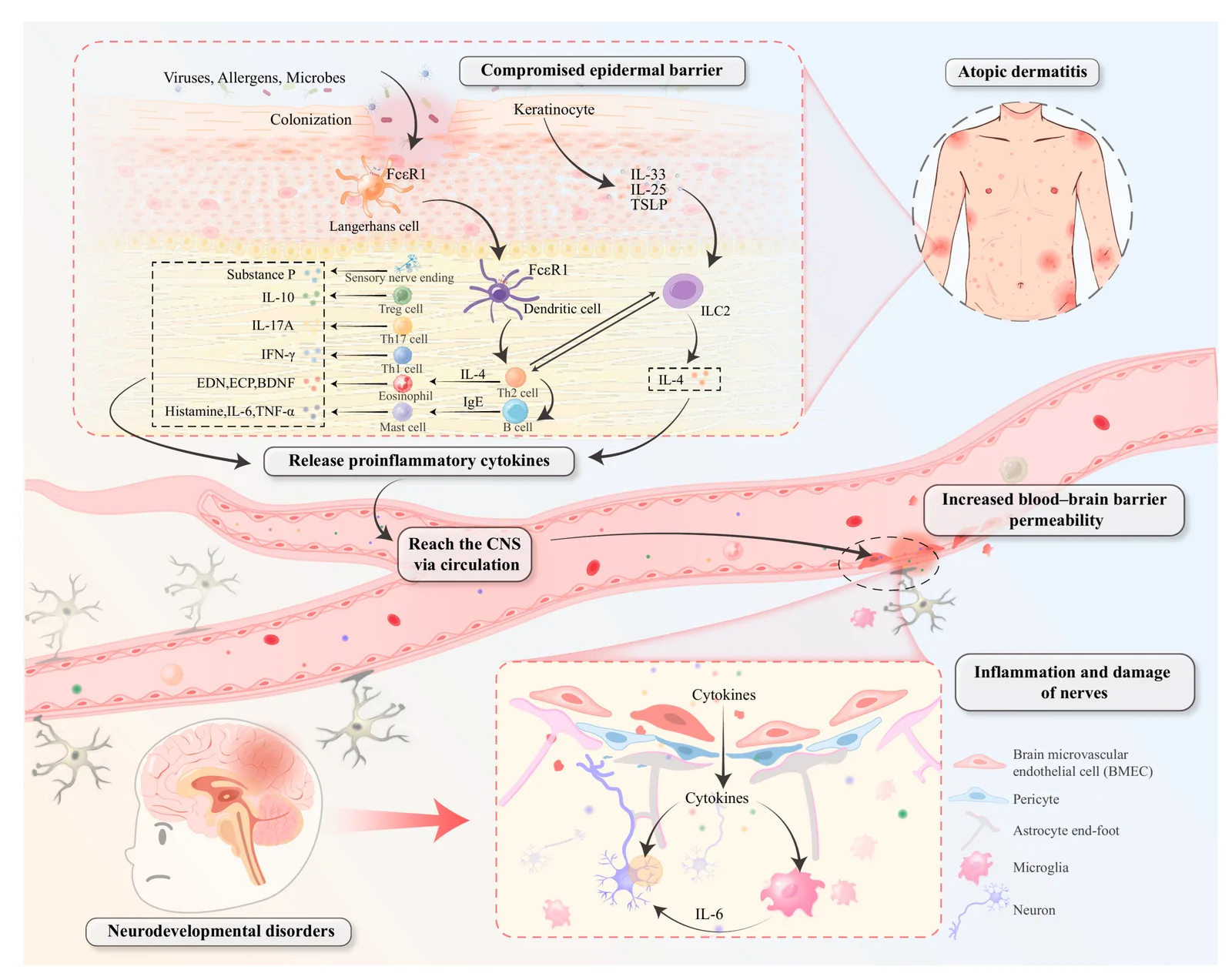
Proposed immune–neural pathways linking atopic dermatitis to neurodevelopmental disorders. Schematic diagram integrating evidence from pediatric and adult human studies, often examining atopic dermatitis (AD) and neurodevelopmental disorders (NDDs) separately, and from cell-based experiments and animal models of AD, maternal immune activation, and other inflammatory or neurological conditions. A compromised epidermal barrier permits exposure to viruses, allergens, and bacteria, triggering responses involving Langerhans cells, dendritic cells, mast cells, eosinophils, and T helper (Th1, Th2, and Th17) cells. Pro-inflammatory cytokines and mediators enter the systemic circulation and may increase blood–brain barrier permeability. These signals may activate microglia, promote neuroinflammation, and affect neuronal function and neurodevelopment. Arrows indicate proposed relationships across these evidence sources. Abbreviations: AD, atopic dermatitis; BBB, blood–brain barrier; BMEC, brain microvascular endothelial cell; CNS, central nervous system; IFN-γ, interferon-γ; IL, interleukin; NDD, neurodevelopmental disorder; Th, T helper; TNF-α, tumor necrosis factor-α; TSLP, thymic stromal lymphopoietin.

Immune dysregulation in AD and in subsets of patients with ASD or ADHD provides a potential mechanistic link between cutaneous and neurodevelopmental pathology (Figure 2). The supporting evidence includes pediatric and adult human studies, often examining AD and NDD populations separately, alongside AD models and other experimental systems. These findings support biological plausibility but do not establish a causal skin–brain pathway in children with both conditions.

#### 4.1.1. Innate Immune Mechanisms

A hallmark of AD is a defective epidermal barrier, often associated with filaggrin (FLG) gene mutations [29]. Barrier dysfunction permits the penetration of allergens, irritants, and microbes, initiating cutaneous inflammation that may provide a peripheral starting point for the proposed skin–brain axis [1,29,30].

Among innate immune cells, mast cells are potential mediators connecting skin inflammation with neural responses. Activated by allergens, neuropeptides, or stress, they release histamine, IL-6 and tumor necrosis factor (TNF)-α, while their proposed effects on the blood–brain barrier (BBB) and microglial activation are based largely on experimental findings [31]. In a prenatal valproic acid (VPA)-exposure mouse model of ASD, increased mast-cell numbers were observed in both skin and brain alongside AD-like skin changes [32,33].

Eosinophils may contribute to this link by releasing neuroactive mediators, including eosinophil-derived neurotoxin (EDN) and eosinophil cationic protein (ECP), both capable of affecting neuronal tissue [34,35,36,37]. They can also produce and secrete brain-derived neurotrophic factor (BDNF) [38,39]. Elevated serum BDNF levels—correlating with AD severity and ECP levels—have been reported in children with AD [34], with elevated serum levels also observed in adult AD cohorts [40,41]. In human AD skin, BDNF-positive eosinophils were observed near nerve fibers, while activated eosinophil supernatants induced BDNF-dependent branching of cultured mouse dorsal root ganglion sensory neurons [38]. Given BDNF’s role in synaptic plasticity and cortical development [42], these findings suggest an eosinophil-mediated immune–neural link in AD. Whether these peripheral changes affect brain BDNF or neurodevelopment in children with AD and an NDD remains unresolved.

In addition, keratinocytes participate in cutaneous immune signaling through the release of IL-25, IL-33 and thymic stromal lymphopoietin (TSLP) in response to barrier disruption [43,44,45,46]. These alarmins drive type 2 immune polarization and sustain inflammation [45,47]. Experimental AD models support TSLP-mediated type 2 immune activation [44]. Interactions involving IL-33, nerve growth factor (NGF), and substance P (SP) have also been proposed to connect peripheral inflammation with neural activation [48,49].

#### 4.1.2. Adaptive Immune Mechanisms

AD involves interactions between innate and adaptive immune cells [50]. T helper (Th) cell-mediated inflammation is central to AD pathogenesis [1,30] and may provide a mechanistic link with NDDs [31,32]. Acute AD lesions exhibit Th2-skewed responses with CD4^+^ T-cell and eosinophil infiltration [1,30,51], whereas chronic disease can also involve Th1 and Th17 activation [1,2,46]. The relative contribution of these pathways varies by age and disease phenotype [52].

Cytokines—including IL-6, TNF-α, IL-1β, IL-17A, and interferon-γ (IFN-γ)—have been implicated in both AD and NDD research involving separate patient populations and experimental models [31,32,52,53,54]. In human cell-based BBB models and mouse models of systemic inflammation or autoimmune encephalomyelitis, inflammatory signaling can compromise BBB integrity and modulate glial activity, thereby promoting neuroinflammatory cascades [55,56,57,58]. IL-6 overexpression alters neuronal adhesion, migration, and synapse formation in cultured mouse cerebellar granule cells [53], while IFN-γ exposure alters neuronal morphology and NDD-associated gene expression in human induced pluripotent stem cell (iPSC)-derived neural cells [54]. Meanwhile, IL-17A—a cytokine elevated in some AD skin profiles [52]—contributes to microglial activation and cognitive impairment in aged rats exposed to lipopolysaccharide (LPS) [59], underscoring its potential neuroinflammatory role.

Distinct Th cell subsets in skin–brain crosstalk:

**Th2 cells.** Th2 cells are pivotal in AD, producing IL-4, IL-5, and IL-13, which drive type 2 inflammation [50,60]. Elevated serum levels of the Th2-associated chemokines macrophage-derived chemokine (MDC/CCL22) and thymus and activation-regulated chemokine (TARC/CCL17) have also been observed in children with ASD without allergic manifestations [61]. Notably, in mice, early postnatal IL-4 elevation following peripheral IL-4 administration or in an allergic asthma model impairs microglia-dependent cerebellar neuronal pruning and leads to hyperactivity and impulsivity resembling ADHD-like behaviors [62].

**Th17 cells.** Th17-mediated inflammation contributes to AD, with its prominence varying across ages and disease phenotypes [52]. In mouse studies, IL-17A produced by Th17 cells and other immune subsets, including meningeal γδ T cells, influences synaptic plasticity and anxiety-like behavior within the central nervous system (CNS) [32]. In mouse models of maternal immune activation, elevated maternal IL-17A signaling disrupts fetal cortical development and induces autism-like behaviors; maternal IL-17A blockade attenuates these effects [63,64,65].

**Treg cells.** Regulatory T (Treg) cells maintain immune tolerance and restrain excessive inflammation in AD by secreting IL-10 and promoting barrier repair [66]. Reduced peripheral Treg levels have been reported in children with ASD [32]. In the BTBR mouse model of ASD, treatment with the peroxisome proliferator-activated receptor δ (PPARδ) agonist GW0742 increased IL-10 and Foxp3 expression in the brain and periphery, accompanied by reductions in inflammatory markers and repetitive behaviors [67].

**Th1 cells.** Th1-related immune alterations have been reported in separate studies of AD and pediatric NDD populations [68,69]. In children with ASD, greater production of pro-inflammatory and Th1-associated cytokines, including IFN-γ, by stimulated peripheral blood mononuclear cells has been associated with impairments in core ASD features and aberrant behaviors [70]. In chronic AD, increased IFN-γ expression has been reported in adult skin and skin-derived mesenchymal stem cell cultures [52,71]. In a mouse model of ASD induced by prenatal valproic acid exposure, elevated IFN-γ levels were also observed in both skin and brain tissues alongside alterations in ceramide composition [33].

### 4.2. Genetic and Epigenetic Mechanisms

The comorbidity between AD and NDDs may involve genetic susceptibility and epigenetic mechanisms that regulate gene expression in response to environmental exposures.

#### 4.2.1. Genetic Susceptibility

Both AD and NDDs exhibit strong genetic predispositions [24]. Evidence from candidate-gene, expression, and functional studies implicates molecules involved in immune regulation and neural development—such as ADRB2, GATA3, MIF, and BDNF—which may constitute molecular links between cutaneous inflammation and neurodevelopmental abnormalities. A GATA3 polymorphism was associated with atopic eczema in one childhood birth cohort [72], whereas findings in children with ASD concern altered GATA3 expression in peripheral T cells [73]. MIF promoter variation was associated with stereotypical behaviors in US and Dutch ASD family samples [74], while MIF-targeted vaccination improved dermatitis in AD mouse models [75]. Functional evidence also includes ADRB2-dependent regulation of cutaneous allergic inflammation in mice [76] and elevated serum BDNF in children with AD [34]; however, the BDNF Val66Met variant was not associated with AD in a German case–control study [77].

Among these, STAT6, a transcription factor central to Th2 differentiation, represents a potential link between immune dysregulation in AD and ASD. Rare germline gain-of-function variants in STAT6 have been linked to severe allergic phenotypes, including AD [78,79]. In children with ASD, reduced numbers of peripheral CD45^+^STAT6^+^ cells have been reported [80], suggesting altered STAT6-associated immune regulation. Together, these disease-specific genetic and cellular findings suggest that STAT6-associated immune regulation may be relevant to AD–ASD comorbidity, but whether the two conditions share STAT6 susceptibility variants remains to be determined.

#### 4.2.2. Epigenetic Mechanisms

Epigenetic regulation alters gene expression without changing the DNA sequence and provides an interface between genetic susceptibility and environmental exposure. Altered DNA methylation profiles have been reported in AD epidermis, partly correlating with the expression of genes involved in epidermal differentiation and innate immune responses [81]. Similar environmentally responsive epigenetic mechanisms have also been implicated in neurodevelopment. In an ASD-focused birth cohort, prenatal bisphenol A exposure was associated with increased CYP19A1 promoter methylation in cord blood collected at birth, prior to subsequent ASD assessment [82].

Non-coding ribonucleic acids (RNAs), particularly microRNAs (miRNAs) such as miR-146a, further modulate immune and inflammatory pathways [24,83]. MiR-146a shows altered expression in AD lesional skin and ASD patient-derived olfactory mucosal stem cells; its effects on neuronal dendritic arborization have been demonstrated in mouse cell cultures [24,83]. These findings suggest a potential regulatory link between cutaneous inflammation and neurodevelopmental processes.

Maternal influences may affect epigenetic regulation during fetal development [82,84]. Factors such as nutrition, pollutant exposure, microbiota composition, psychological stress, and immune activation may influence offspring immune and neural function through epigenetic and other biological pathways [85,86,87,88,89,90,91,92]. In particular, maternal immune activation (MIA) may influence fetal neurodevelopment through cytokine signaling at the maternal–placental–fetal interface [64]. In MIA mouse models, placental IL-6 signaling mediates fetal brain inflammation and subsequent behavioral abnormalities [86], while maternal IL-17A signaling contributes to altered fetal cortical development and autism-like behaviors [63,64,65]. These findings concern prenatal inflammatory exposure, which is distinct from postnatal inflammation in children with AD. The AD–NDD association may therefore involve shared prenatal determinants and inherited susceptibility, alongside potential postnatal inflammatory effects [24,88,93].

### 4.3. Other Possible Hypotheses

Sleep disturbance, psychosocial stress, and BBB dysfunction provide additional routes through which AD and neurodevelopmental outcomes may be linked, and may interact with the immune pathways described above.

**Sleep disturbance.** Chronic nocturnal pruritus is among the most burdensome symptoms of AD and can contribute to sleep fragmentation [94,95]. Sleep disruption may affect neurocognitive function and exacerbate attention and behavioral problems typical of NDDs [95]. Altered neuroimmune signaling and endocrine rhythms, described in adult sleep studies, are potential mechanisms through which sleep disturbance may amplify the effects of AD on neurocognitive function [95].

**Psychosocial stress and emotional dysregulation.** Psychological stress can precipitate AD flares and engage systemic stress pathways (for example, the hypothalamic–pituitary–adrenal axis) [96]. In adults with AD, acute psychosocial stress increased circulating CD8^+^ T lymphocytes and eosinophils [97]. In children with both AD and ADHD, salivary cortisol responses to acute psychosocial stress were reduced compared with healthy controls, whereas no corresponding reduction was observed in children with AD alone [98]. Emotional and behavioral problems have been associated with AD in pediatric observational studies [28,99] and may further entrench bidirectional skin–brain interactions through increased psychological stress [96].

**Blood–brain barrier dysfunction.** In a mouse model of repeated systemic LPS exposure, peripheral inflammation can compromise BBB integrity, facilitating the entry of peripheral immune cells into the central nervous system with accompanying microglial activation [56]. In an MC903-induced mouse model of AD, BBB disruption was accompanied by glial activation and cognitive deficits; IL-17A blockade attenuated these changes [100]. These experimental findings support BBB dysfunction as a plausible route by which peripheral cutaneous inflammation may contribute to central neuroinflammation with potential implications for neurodevelopment [31].

## 5. Identification of High-Risk Children

Dermatologists managing pediatric AD should ask about developmental, behavioral, and sleep concerns, particularly in children with clinical features associated with higher odds or rates of NDDs. These features may help prioritize developmental surveillance and multidisciplinary referral when indicated (Table 2):

**Disease severity:** moderate-to-severe AD is associated with significantly higher odds of ADHD, potentially reflecting a greater systemic inflammatory load and neuroimmune dysregulation [4]. Regular assessment using validated tools such as Scoring Atopic Dermatitis (SCORAD) or the Eczema Area and Severity Index (EASI) is recommended for tracking skin disease severity [2], and severe AD should prompt closer developmental surveillance, with multidisciplinary evaluation when developmental or behavioral concerns are identified.

**Multiple atopic comorbidities:** coexisting asthma, allergic rhinitis, or conjunctivitis is associated with higher NDD risk—potentially reflecting cumulative allergic inflammation—and merits closer developmental surveillance [17,18].

**Severe or chronic sleep disturbance:** persistent nocturnal pruritus and fragmented sleep warrant assessment of attention and behavior, as the AD–ADHD association is stronger in children with sleeping problems [101]. Sleep disruption may impair cognitive and emotional functioning, supporting active management of itch and sleep difficulties to improve sleep and daytime functioning [95].

**Early age of onset:** AD manifesting before one year of age, particularly with severe or respiratory atopic disease, is associated with heightened ADHD and ASD risk [17]. Early childhood (<5 years) represents a crucial window for age-appropriate neurodevelopmental screening, and dermatologists should coordinate with pediatric clinicians to support adherence to pediatric guidelines for developmental surveillance and ASD-specific assessment [102,103].

For children aged 16–30 months, the Modified Checklist for Autism in Toddlers, Revised with Follow-up (M-CHAT-R/F; 20 items, <5 min for the initial questionnaire) can be used for ASD screening, with structured follow-up when indicated [104,105]. From 24 months, the preschool version of the Strengths and Difficulties Questionnaire (SDQ) for ages 2–4 years can additionally screen for emotional and behavioral difficulties [106,107].

For children older than 30 months and up to 17 years, age-appropriate SDQ versions can screen for emotional and behavioral difficulties, including hyperactivity/inattention (25 items, 5–10 min) [106,107]. When ASD concerns arise, the age-appropriate Social Responsiveness Scale, Second Edition (SRS-2; 65 items, 15–20 min), can help identify ASD-related social difficulties [108]. Positive screens or persistent concerns should prompt further clinical assessment and appropriate referral.

## 6. Clinical Management Strategies

Management of pediatric AD, particularly when NDDs co-occur, should combine effective skin disease control with developmental surveillance and multidisciplinary care. Prenatal findings provide additional context for maternal health and potential preventive research.

### 6.1. Maternal Health During Pregnancy

Adequate prenatal nutrition is essential for fetal neurodevelopment [109]. A balanced diet should be encouraged, and supplementation with folic acid and iron should follow established antenatal recommendations [110]. Observational studies have associated prenatal multivitamin use with lower odds of ASD with intellectual disability [111] and maternal consumption of ultra-processed foods with higher offspring ADHD symptom scores [112]. Trials of vitamin D_3_ and omega-3 fatty acids suggest potential benefits for some early allergic outcomes [113,114].

Beyond nutrition, exposure to smoking should be avoided [110]. Prenatal air pollution exposure has been associated with offspring ASD, providing a rationale for reducing avoidable exposure [115]. Management of maternal health conditions—such as thyroid dysfunction, polycystic ovary syndrome (PCOS), and psychological stress—remains important in antenatal care. Maternal thyroid dysfunction and PCOS have been associated with offspring NDDs [116,117], while prenatal stress has been associated with childhood atopic diseases [118].

### 6.2. Targeted Immune Therapy for Moderate-to-Severe AD in Children

Biologics targeting IL-4/IL-13 signaling, including dupilumab, tralokinumab, and lebrikizumab, have expanded treatment options for moderate-to-severe AD [1,119,120,121]. Beyond cutaneous control, a population-based pediatric cohort associated dupilumab with lower risks of new-onset anxiety, mood disorders, ADHD, and sleep disturbances compared with other systemic agents [122]. A separate retrospective cohort associated dupilumab treatment with decreased psychostimulant use in patients with AD and ADHD [123]. Relief of pruritus, improved sleep and quality of life, and reduced inflammation may contribute to these observations.

JAK inhibitors, such as upadacitinib, can also rapidly relieve pruritus in adolescents with moderate-to-severe AD through inhibition of JAK1-dependent cytokine signaling [124,125]. Long-term safety requires continued assessment in pediatric populations. Symptom relief and lower rates of recorded diagnoses or medication use do not establish modification of neurodevelopment. Prospective studies combining repeated measures of pruritus, sleep, quality of life, and inflammatory biomarkers with standardized neurodevelopmental assessments and mediation analyses could clarify the contributions of symptom relief and immune modulation.

### 6.3. Multidisciplinary Management

A multidisciplinary approach involving dermatology, pediatrics, and psychological or nutritional services as needed can address the varied burden of AD and its comorbidities. Standard dermatologic care includes regular emollient use to support the skin barrier and topical corticosteroids for flares [1,2]. Topical calcineurin inhibitors (TCIs) are useful for sensitive sites, phosphodiesterase-4 (PDE-4) inhibitors offer an additional topical option, and wet-wrap therapy can be an adjunct for moderate-to-severe disease [1,2]. Developmental or psychological concerns should prompt timely assessment and referral [126]. Psychological interventions should match the presenting difficulties: cognitive behavioral therapy (CBT) and mindfulness-based approaches may support attention and behavior in children with ADHD [127], while CBT can reduce co-occurring anxiety in children with ASD [128]. Adult AD trials have reported improvements in eczema symptoms or quality of life with CBT and mindfulness-based interventions [129,130]. Sleep hygiene education and age-appropriate pruritus management may also reduce sleep disturbance [1,2].

Coordination across preconception, pregnancy, and childhood can bring together nutritional, environmental, and psychological approaches to support development [131]. Microbiome-targeted approaches, including probiotics and experimental fecal microbiota transplantation (FMT), are being investigated for AD control [132]. Small studies in ADHD populations have reported potential benefits of probiotics for selected symptoms or quality-of-life outcomes [133]. These findings warrant further evaluation in children with co-occurring AD and NDDs.

## 7. Controversies and Limitations

Epidemiologic associations between childhood AD and NDDs vary across populations, and several studies have reported non-significant findings for ASD or ADHD [19,22,134,135]. Both clinical and methodological heterogeneity may contribute. Clinically, AD is not a uniform exposure: age at onset, severity, persistence, symptom burden, and atopic multimorbidity may distinguish subgroups with different neurodevelopmental risk profiles. Pooling transient or mild AD with early-onset, persistent, or severe disease may obscure subgroup associations, but the available evidence does not establish phenotype-specific causal pathways.

Methodological heterogeneity includes differences in diagnostic criteria, healthcare systems, AD ascertainment, exposure and outcome timing, severity and disease-course characterization, and confounder adjustment. These differences limit comparability across studies. Residual confounding, reverse causation, and ascertainment bias remain concerns in observational research. Meta-analyses may also contain overlapping primary studies, so agreement between pooled estimates should not automatically be interpreted as independent replication.

Mechanistic evidence is also inconclusive. Conflicting findings concern maternal inflammation and offspring ADHD risk [136,137], immune-cell alterations in ASD [138], and the relationship between AD severity and cognitive function in adults [139]. Immune mediators can exert context-dependent effects: in children with ASD, selected stimulated immune responses, including GM-CSF and some Th2-related cytokines, were associated with better cognitive or adaptive outcomes [70]. A bidirectional Mendelian randomization study also reported differing associations between specific immune-cell traits and ADHD [140]. These observations suggest that immune effects vary with developmental stage, cellular context, tissue compartment, and disease state. Genetic evidence is inconsistent; for example, the proposed role of BDNF in immune and neural processes has not translated into a significant association between the BDNF Val66Met variant and AD [77]. Epigenetic findings likewise differ by tissue and sampling time: AD epidermal methylation was assessed in established disease [81], whereas cord-blood methylation was measured before later ASD assessment [82]. Inflammation, treatment, and altered cell composition may influence epigenetic profiles in established disease, complicating the distinction between antecedent susceptibility and disease-related changes. The proposed skin–brain axis therefore likely involves interacting immune, genetic, neuroendocrine, and behavioral pathways rather than a single linear mechanism.

Therapeutic evidence remains limited. Prenatal vitamin D studies have yielded heterogeneous findings for subsequent allergic outcomes [113,141], and evidence for probiotics in ADHD is preliminary and inconsistent [133]. Variation in populations, intervention timing, dosage, and outcome definitions may contribute. Associations between dupilumab and neuropsychiatric outcomes currently derive from observational studies [122,123], leaving their causal interpretation unresolved.

Future research should test the temporal and mechanistic relationships underlying AD–NDD associations. Prospective cohorts should distinguish AD onset, severity, persistence, and atopic multimorbidity, integrating biomarkers, immune profiles, sleep measures, and standardized neurodevelopmental assessments. Sex-related differences in diagnostic recognition, including potential under-recognition of ADHD and ASD in girls, may influence observed comorbidity patterns [142,143]. Prespecified sex-stratified analyses, formal tests of sex-by-AD interactions, and comparable assessments in boys and girls would help clarify these patterns. Biomarker- and endotype-based stratification may identify susceptible subgroups. Prospective treatment studies should determine whether early control of AD inflammation, pruritus, and sleep disturbance can modify long-term neurodevelopmental trajectories.

## 8. Conclusions

Pediatric AD is associated with NDDs, particularly ADHD and ASD, with stronger associations reported in severe or early-onset disease. Immune dysregulation, potential genetic and epigenetic links, sleep disturbance, and psychosocial stress may contribute to the proposed skin–brain connection, although causality remains uncertain. Dermatologists should remain alert to developmental, behavioral, and sleep concerns, particularly in children with higher-risk clinical features, and facilitate age-appropriate assessment and multidisciplinary referral when indicated. Effective control of skin inflammation, pruritus, and sleep disturbance remains central to care. Whether early targeted AD treatment can modify neurodevelopmental trajectories requires prospective investigation.

## Figures and Tables

**Table 1:** Meta-analytic evidence linking atopic dermatitis with neurodevelopmental and mental-health outcomes.

Disorder	Source, Year	Included Studies (Participants)	Pooled Effect (95% CI)	Heterogeneity	Key Finding
ADHD	Wang et al. (2025) [4]	36 studies (*n* = 7,118,647)	OR 1.34 (1.25–1.44)	I^2^ = 86.8%	AD and ADHD are associated; estimates are stronger with greater AD severity and comorbidities.
ADHD	Wang et al. (2025) [4]	36 studies (*n* = 7,118,647)	HR 1.42 (1.20–1.68)	I^2^ = 97%	AD and ADHD are associated; estimates are stronger with greater AD severity and comorbidities.
ADHD	Cheng et al. (2024) [7]	19 studies (*n* = 71,005,269)	OR 1.28 (1.18–1.40)	I^2^ = 85%	AD is associated with significantly higher odds of ADHD.
ADHD	Xie et al. (2019) [6]	35 studies (*n* = 2,068,911)	OR 1.563 (1.382–1.769)	I^2^ = 92.53% *	AD is associated with higher odds of ADHD.
ADHD	Schans et al. (2017) [5]	6 studies (*n* > 226,550)	OR 1.32 (1.20–1.45)	I^2^ = 0% (*p* = 0.64)	AD is associated with significantly higher odds of ADHD.
ADHD	Miyazaki et al. (2017) [11]	5 studies (*n* = 61,811)	OR 1.43 (1.09–1.88)	I^2^ = 87%	Children with ADHD have higher odds of atopic dermatitis.
ASD	Elgenidy et al. (2025) [12]	30 studies overall; 23 in the ASD-to-eczema analysis	RR 1.34 (1.03–1.76)	I^2^ = 99%	ASD is associated with a higher risk of atopic eczema.
ASD	Cheng et al. (2024) [7]	3 studies (*n* = 1,265,137)	OR 1.87 (1.30–2.68)	I^2^ = 84%	AD is associated with higher odds of ASD.
ASD	Xie et al. (2019) [6]	35 studies (*n* = 2,068,911)	Children: OR 2.574 (1.469–4.510)	I^2^ = 92.53% *	AD is associated with higher odds of ASD.
Depression	Xie et al. (2019) [6]	35 studies (*n* = 2,068,911)	Children: OR 1.402 (1.256–1.565)	I^2^ = 92.53% *	AD is associated with higher odds of depression.
Depression	Rønnestad et al. (2018) [10]	23 studies (*n* = 6,587,298)	Adults: OR 2.19 (1.87–2.57); Children: OR 1.27 (1.12–1.45)	I^2^ > 75% *	AD is associated with higher odds of depression.
Depression	Patel et al. (2019) [8]	36 studies (Not reported)	OR 1.71 (1.48–1.98)	I^2^ > 90%	AD is associated with higher odds of depression.
Anxiety	Xie et al. (2019) [6]	35 studies (*n* = 2,068,911)	Children: OR 1.339 (1.062–1.687)	I^2^ = 92.53% *	AD is associated with higher odds of anxiety.
Anxiety	Rønnestad et al. (2018) [10]	13 studies (*n* = 4,641,261)	Adults: OR 2.19 (1.75–2.73)	I^2^ > 75% *	AD is associated with higher odds of anxiety.
Suicidal ideation	Rønnestad et al. (2018) [10]	6 studies (*n* = 94,445)	Adults: OR 4.32 (1.93–9.66)	I^2^ > 75%	AD is associated with higher odds of suicidal ideation.
Suicidal ideation	Patel et al. (2019) [8]	14 studies (Not reported)	OR 1.97 (1.19–3.25)	I^2^ > 90%	AD is associated with higher odds of suicidal ideation.
Suicidal ideation	Sandhu et al. (2019) [9]	11 studies (*n* = 4,770,767)	OR 1.44 (1.25–1.65)	I^2^ = 8%	AD is associated with higher odds of suicidal ideation.
Suicide attempts	Sandhu et al. (2019) [9]	3 studies (*n* = 4,770,767)	OR 1.36 (1.09–1.70)	I^2^ = 80%	AD is associated with higher odds of suicide attempts.

Data are presented as pooled ORs, HRs, or RRs with 95% CIs. Asterisks (*) indicate heterogeneity reported for broader analyses in the source reviews rather than for the specific outcome or age subgroup shown. For Xie et al. [6], I^2^ = 92.53% refers to the overall analysis of total mental disorders. Abbreviations: AD, atopic dermatitis; ADHD, attention-deficit/hyperactivity disorder; ASD, autism spectrum disorder; CI, confidence interval; HR, hazard ratio; I^2^, heterogeneity statistic; OR, odds ratio; RR, risk ratio.

**Table 2:** Clinical features associated with increased neurodevelopmental risk in children with AD.

Clinical Feature	Comparison Group	ADHD Effect Measure (95% CI)	ASD Effect Measure (95% CI)	Clinical Implications and Recognition Points
Greater AD severity	ADHD: severe/mild-to-moderate AD vs. no AD. ASD: ≥4/1 AD visits before age 2 years vs. no AD before age 2 years.	Severe: pooled OR 2.62 (1.76–3.92). Mild-to-moderate: pooled OR 1.33 (1.10–1.60) [4].	≥4 visits: aHR 1.19 (1.06–1.34). 1 visit: aHR 1.09 (1.01–1.18) [17].	Use EASI or SCORAD to document AD severity; greater severity may warrant closer developmental surveillance.
Multiple allergic comorbidities	Infantile AD alone or with subsequent AD episodes and ARD, vs. no corresponding atopic exposures.	AD alone: aHR 1.16 (1.13–1.19). With subsequent AD episodes and ARD onset at ages 2–5 years: aHR 2.05 † [17].	AD alone: aHR 1.10 (1.03–1.18). With subsequent AD episodes and ARD onset at age ≥ 6 years: aHR 2.13 † [17].	Assess coexisting asthma or allergic rhinitis and associated developmental or behavioral concerns.
Sleep disturbance	AD vs. no AD, stratified by sleep problems; ages 3–11 years.	With sleep problems: adjusted OR 2.67 (1.51–4.71). Without: adjusted OR 1.24 (0.83–1.84) [101].	—	Ask about night awakenings and difficulty initiating sleep; address sleep disturbance and assess daytime attention and behavior.
Early-onset AD	Onset before age 1 year or at ages 1–2 years, vs. no AD before age 2 years.	<1 year: aHR 1.17 (1.13–1.20). 1–2 years: aHR 1.14 (1.09–1.18) [17].	<1 year: aHR 1.11 (1.04–1.20). 1–2 years: aHR 1.07 (0.98–1.18) [17].	Early-onset AD warrants age-appropriate developmental surveillance, particularly with persistent or complex disease.

ORs and aHRs describe relative associations, not absolute risk increases. Subgroup estimates refer to the stated reference groups and do not represent direct comparisons between subgroups. ADHD severity estimates [4] were pooled across age groups; ASD severity estimates [17] used AD-related visit frequency before age 2 years as a proxy for severity. † The combined-exposure estimates are presented with graphical 95% CIs in the original study, but numerical confidence limits are not reported [17]. Abbreviations: AD, atopic dermatitis; ADHD, attention-deficit/hyperactivity disorder; aHR, adjusted hazard ratio; ARD, allergic respiratory disease; ASD, autism spectrum disorder; CI, confidence interval; EASI, Eczema Area and Severity Index; OR, odds ratio; SCORAD, Scoring Atopic Dermatitis.

## Data Availability

This article synthesizes previously published studies and generates no new datasets. All data cited are available in the published articles listed in the references.
